# Effect of Intranasal Calcitonin in a Patient with McCune-Albright Syndrome, Fibrous Dysplasia, and Refractory Bone Pain

**DOI:** 10.1155/2017/7898713

**Published:** 2017-06-06

**Authors:** Tayane Muniz Fighera, Poli Mara Spritzer

**Affiliations:** ^1^Gynecological Endocrinology Unit, Division of Endocrinology, Hospital de Clinicas de Porto Alegre, Rua Ramiro Barcelos 2350, 90035-003 Porto Alegre, RS, Brazil; ^2^Laboratory of Molecular Endocrinology, Department of Physiology, Federal University of Rio Grande do Sul, Rua Ramiro Barcelos 2350, 90035-003 Porto Alegre, RS, Brazil

## Abstract

McCune-Albright syndrome (MAS) is a rare disease defined by the triad of polyostotic fibrous dysplasia of bone, café-au-lait skin spots, and precocious puberty. No available treatment is effective in changing the course of fibrous dysplasia of bone, but symptomatic patients require therapeutic support to reduce bone pain and prevent fractures and deformities. We report the case of a 27-year-old woman with MAS and severe fibrous dysplasia. She was diagnosed with MAS at 4 years of age and, during follow-up, she had multiple pathological fractures and bone pain refractory to treatment with bisphosphonates, tricyclic antidepressants, and opioids. The pain was incapacitating and the patient required a wheelchair. Intranasal calcitonin was then started, and, 30 days later, the patient already showed significant improvement in pain severity at the affected sites. After 3 months, she was able to walk without assistance. No adverse effects were observed, nor were any significant changes in serum levels of calcium, phosphorus, and alkaline phosphatase. Calcitonin has a well-recognized analgesic effect on bone tissue. Despite the small number of studies involving patients with MAS, calcitonin may be considered a short-term therapeutic option in cases of severe and refractory bone pain.

## 1. Introduction

McCune-Albright syndrome (MAS) is classically defined by the presence of fibrous dysplasia (FD),* café-au-lait* skin pigmentation, and precocious puberty. Other hyperfunctioning endocrinopathies may be involved, including hyperthyroidism, growth hormone excess, Cushing syndrome, and renal phosphate wasting. MAS has an estimated prevalence ranging from 1/100.000 to 1/1000.000, affects both sexes equally, and is generally diagnosed in children and young adults [1].

Patients with MAS have involvement of multiple bone sites (polyostotic FD) that is usually established early in life [2]. FD occurs when bone marrow cells are affected by somatic activating mutations of the gene encoding the *α*-subunit of the stimulatory G protein (Gs *α*). The mutation results in locally increased stimulation of adenylyl cyclase and cAMP overproduction, leading to autonomic secretion in endocrine tissues. At the bone tissue level, FD is characterized by dysplastic lesions consisting of abnormal and poorly organized fibrous tissue, with a lytic or cystic appearance [3]. The natural course of bone disease is highly variable. Lesions can remain stable for decades, but they can also progress to multiple fractures and severe bone pain and deformities, which can be extremely debilitating [1, 2].

Clinical studies on FD are difficult because this condition is rare and clinically heterogeneous. There are no available medical therapies capable of altering the disease course. Recently, a multidisciplinary workshop, including patients, clinicians, and researchers, discussed the priorities of diagnosis and treatment of patients with FD and MAS. Among these priorities is the management of chronic pain with typical and atypical analgesics as well as adjuvant interventions when necessary [4]. The primary target of treatment may be the relief of bone pain and reduction of fracture risk and deformity. Intravenous bisphosphonates, such as zoledronic acid and pamidronate, may be effective in reducing bone pain and bone resorption as well as in improving the radiographic appearance of lytic lesions. Calcium and vitamin D supplementation may also be considered [5]. However, some patients have poor response to available therapies.

We report here a case of MAS presenting severe FD and refractory bone pain and the effect of short-term treatment with intranasal calcitonin, highlighting the challenges of the management of this uncommon clinical presentation.

## 2. Case Presentation

A 4-year-and-8-month-old girl was referred to the endocrinology outpatient clinic in 1993 for evaluation of bilateral development of breast tissue followed by vaginal bleeding lasting 5 days. After 2 months, she had another episode of vaginal bleeding, with duration similar to that of the first episode. She was born from vaginal delivery at term, weighed 2,350 kg, and had adequate motor and cognitive development. She used no continuous medication. On physical examination, the patient had a weight of 15,5 kg (p25), height of 1,02 m (p25), Tanner stage M1P1, and absence of café-au-lait skin pigmentation. Bone age was compatible with chronological age. Pelvic ultrasound showed uterus with 3.2 cc, endometrium of 0.2 cm, right ovary with a volume of 0.5 cc, and left ovary with a cyst measuring 1.5 × 2.3 cm. Laboratory tests showed estradiol 18.3 pmol/L, LH 1.70 IU/L, FSH 2.10 IU/L, prolactin 15 *μ*g/L, cortisol 400 nmol/L, TSH 1.3 mIU/L, and a prepubertal response to GnRH test. An initial diagnosis of autonomous ovarian follicular cyst was then made and the patient was kept on regular clinical follow-up with expectant management. After 7 months of follow-up, progression of premature thelarche (M2P1) was observed, with serum estradiol levels 38.2 pmol/L and normal prepubertal gonadotropin levels. Bone scintigraphy showed increased radioisotope concentration in the humerus, femur, tibia, and maxilla on the right side. Bone densitometry showed adequate bone mineral density (BMD) for age. The 24 h urine analysis showed a tubular phosphorus reabsorption rate of 88%, with phosphaturia of 16.9 mmol/24 h. Due to the presence of precocious puberty and FD, the patient was diagnosed with MAS.

**Figure 1:**
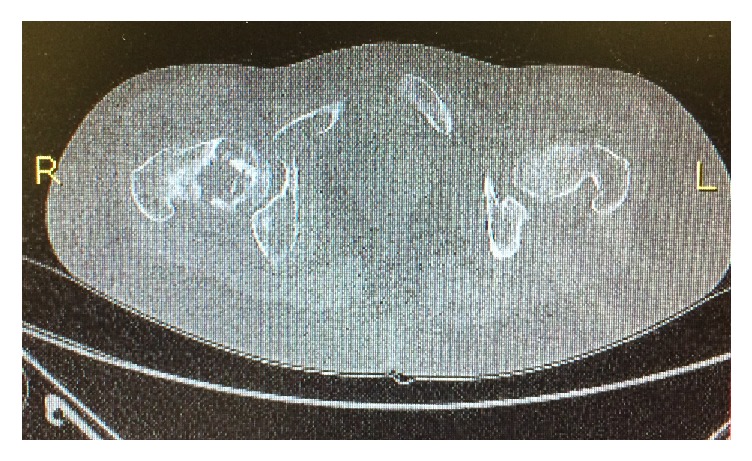
Computed tomography at age 17. Osteolytic lesions and areas of “ground-glass” opacity in the right femur.

**Figure 2:**
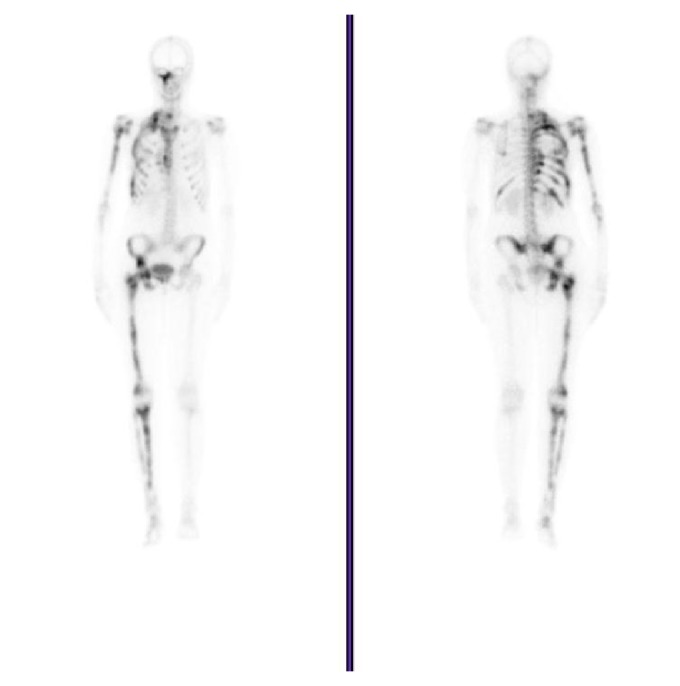
Bone scintigraphy at age 26. Diffusely increased osteoblastic activity on the right side of the body and tenth left costal arch.

**Figure 3:**
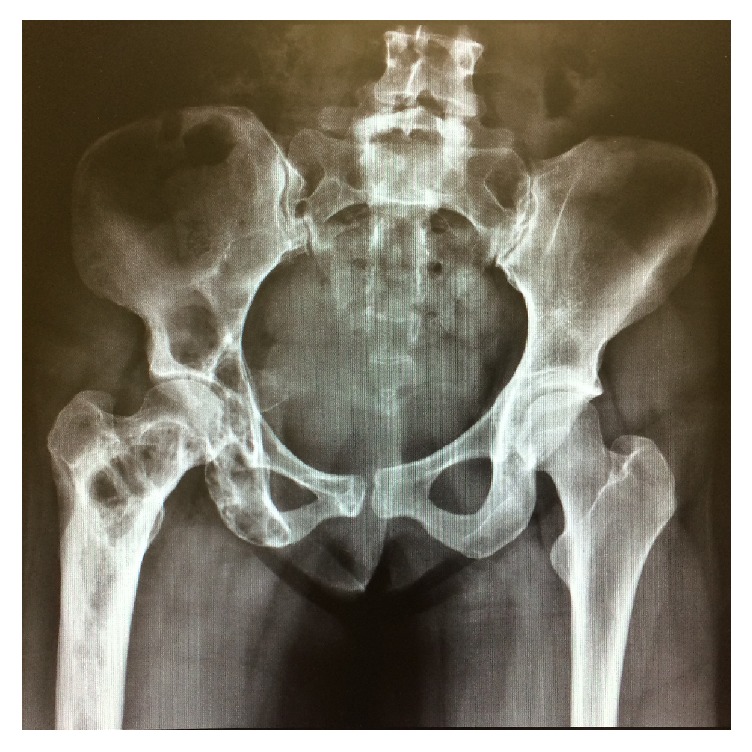
Hip Rx at age 27. Lesions in the iliac bone and right femur compatible with fibrous dysplasia.

At that time, letrozole was not yet available in the country and suppressive therapy was started with 200 mg of intramuscular medroxyprogesterone every 3 weeks, with regression of breast tissue. A bone age X-ray performed at follow-up was compatible with chronological age. No increase in ovarian volume was detected on pelvic ultrasound. She had menarche at age 11, with irregular cycles and facial acne that improved with oral contraceptives. At age 14, due to bone pain in the right thigh and radiographic evidence of cysts, the patient started receiving a treatment protocol with intravenous pamidronate every 6 months, 40 mg/day for 3 consecutive days, for five cycles. The dose was not increased because of patient intolerance to medication. Over the follow-up period, vitamin D levels were monitored and supplementation provided if necessary. Treatment response was assessed by subjective pain intensity, alkaline phosphatase levels, and serial bone scintigraphy. At age 17, the patient had severe spontaneous pain in the hip, and a computed tomography scan showed interruption of the cortex in the right femoral neck related to fracture (Figure 1). The patient then underwent local curettage followed by a lyophilized bovine bone grafting in the right proximal femur. Bone tissue biopsy showed areas of fibrosis and hyalinization, associated with immature trabecular bone, compatible with the diagnosis of FD. Two years after this procedure, the patient had a costal arch fracture after minimal trauma. During the past year, there was progressive worsening of pain, especially in the hip region, refractory to different analgesic regimens that included tricyclic antidepressants and opioids. Bone scintigraphy revealed diffusely increased osteoblastic activity in the right hemibody (Figure 2). Bone densitometry showed *Z*-score −0.7 in lumbar spine (BMD 1.091 g/cm^2^), −1.3 in left total femur (BMD 0.840 g/cm^2^), and −2.8 in forearm 33% (BMD 0.629 g/cm^2^). The patient had severe pain, according to a visual scale of pain [6], and had great difficulty in walking and required a wheelchair, even at home. An Rx showed lesions compatible with fibrous dysplasia (Figure 3). She was seen by an orthopedist, who suggested conservative treatment, with no indication for further surgery. A new cycle of pamidronate, with 160 mg divided into 3 days (40 mg on the first day and 60 mg on the second and third days), produced no improvement. After obtaining written informed consent, we then started calcitonin administered as a nasal spray 200 UI once daily. The patient returned 2 weeks later reporting good tolerance and significant improvement in pain severity. The dose was increased to 200 UI twice daily and the patient returned after 30 days of treatment walking with the aid of crutches but without a wheelchair. She no longer needed opioids every day, which had a significant impact on quality of life, since the patient was intolerant of this class of medications. Three months after the start of nasal calcitonin, the patient is able to walk without assistance, with mild pain, estimated by the visual scale in the hip and sporadic opioid use. While no specific biochemical markers of bone turnover, such as amino-terminal propeptide (PINP) of type I collagen and carboxy-terminal collagen crosslinks (CTX), were available for this patient, serum alkaline phosphatase, an unspecific bone turnover marker, and calcium and phosphorus levels were assessed and did not change during treatment (Table 1).

## 3. Discussion

The present report shows the favorable outcome of a patient with MAS and severe bone pain after short-term treatment with nasal calcitonin. Calcitonin is a 32-amino acid polypeptide hormone produced by the parafollicular cells of the thyroid gland whose secretion is mainly regulated by serum calcium levels. Its main role is to inhibit bone resorption by reducing osteoclast activity [3].

FD is characterized by the development of fibrous bone lesions that replace normal skeletal structures. Abnormal fibroblast proliferation and defective osteoblast differentiation result in the replacement of cancellous bone and marrow with fibrous connective tissue. There is no cure or spontaneous resolution of FD, but, since bone pain, deformities, and pathological fractures are the main symptoms, the condition often requires treatment [7]. However, because it is a rare disease, few data are available in the literature addressing these concerns.

Benhamou et al. [8] recently conducted an analysis of 372 patients with FD, 42% of whom were diagnosed with a polyostotic form and 12% with MAS. The main symptom at diagnosis was bone pain, which occurred in 44% of patients, followed by fracture in 9%. In univariate analysis, younger age at diagnosis, renal phosphate wasting, a polyostotic form of FD, fracture, and bisphosphonate use were significant predictors. In the multivariate model, the polyostotic form and bisphosphonate use remained significant predictors. However, those who were treated were likely to have more severe disease.

Bisphosphonates are often used as a medical treatment to reduce the increased bone turnover in the affected bone tissue. Thomsen and Rejnmark [7] published a review on the treatment of 26 cases of FD, 4 of which were diagnosed with MAS. Most patients received bisphosphonate treatment (89%), but it did not result in significant relief of symptoms or radiological improvement of the lesions. The mean duration of treatment was 4 years (3–276 months), and the types of bisphosphonates prescribed changed during follow-up. Only 3 patients reported pain relief with treatment. Boyce et al. (2014) also evaluated 35 patients with FD with at least 2 skeletal lesions. Alendronate was administered over a 24-month period in 6-month cycles, with stratified doses by weight. There was no difference in mean pain score and functional tests between alendronate and placebo groups at any point during the treatment period [9].

The use of calcitonin in patients with FD is not new. The first study published by Bell et al. [10] in 1970 investigated the effects of calcitonin in 5 patients, 4 with a diagnosis of Paget's disease and 1 with polyostotic FD. Calcitonin was given intramuscularly every 12 hours for 16 days, with the patients hospitalized under medical care. No significant changes were observed in serum levels of calcium, phosphorus, and alkaline phosphatase. Calcitonin reduced calciuria in 3 patients and fecal calcium in all patients, but these changes occurred only during treatment. Urinary hydroxyproline levels decreased significantly in 2 patients and did not increase again even 30 days after the end of treatment. Similar findings were described by Yamamoto et al. [11] in a 12-year-old girl with a diagnosis of MAS. In this case, treatment with a synthetic analog of calcitonin administered twice weekly for 20 weeks led to a progressive reduction in urinary proline and hydroxyproline levels. Other study has not confirmed this effect of calcitonin on bone turnover markers [12].

The analgesic activity of calcitonin has been demonstrated in several trials in patients suffering from different painful skeletal conditions [13–15]. The mechanism of the analgesic effect of calcitonin remains unclear, but some evidence for its role in decreasing pain has already been described [16]. Indeed, calcitonin-binding sites have been detected in the hypothalamus and other areas of the central nervous system and seems to depend on the integrity of the serotonergic pathway [17]. In addition, calcitonin possibly inhibits the production of prostaglandins and other proinflammatory cytokines, through a reduction in cyclogenase activity [18]. It also induces a reduction of calcium influx in the neural membrane, which makes the target cells less reactive and decreases the stimulation of nociceptors located in the synovia and periosteum [18, 19]. Other putative mechanisms include elevated plasma *β* endorphin levels and effects on central serotonergic or monoaminergic pathways [13]. Although antibody formation against human calcitonin is rare, approximately 40 to 70% of patients receiving long-term therapy with salmon calcitonin produce specific antibodies. The clinical significance of these antibodies is unclear; however, clinical trials in postmenopausal osteoporosis have shown that these antibodies do not reduce the efficacy of long-term treatment [3].

Concerning potential adverse effects of calcitonin, the European Medicines Agency (EMA) recently published a press release stating that the increase in cancer rates with calcitonin varied between 0.7% in studies with the oral formulation and 2.4% in the studies with the nasal formulation [20]. However, the EMA did not advise against short-term use (less than 3 months), especially in patients with Paget's disease, bone loss associated with immobilization, and cancer related hypercalcemia. In addition, studies with fracture-related bone pain have shown benefit of the analgesic properties of calcitonin when used for short term, but not for long periods [21, 22]. In a meta-analysis of 13 studies in patients with osteoporotic vertebral compression fractures, there was a significant reduction of bone pain with onset in less than 10 days, with continued improvement through 4 weeks. For patients with pain for more than 3 months, there was no significant improvement [22]. Another study evaluated 91 patients with breast cancer and anastrozole-induced bone pain. The results showed a significant reduction of pain in women receiving calcitonin for three months when compared to the control group [23].

We reported the case of a patient with a diagnosis of MAS and incapacitating bone pain refractory to treatment with intravenous bisphosphonate associated with opioids and tricyclic antidepressants. There are only a few references in the literature to the use of calcitonin in MAS, but, in the present case, there was a significant improvement in both bone pain severity and quality of life. The treatment was very well tolerated and no adverse effects were noted. Based on this experience, short-term use of calcitonin may be considered an effective alternative in selected patients with polyostotic FD and severe and refractory bone pain.

## Figures and Tables

**Table 1:** Bone metabolism evaluation.

	Before calcitonin treatment	During calcitonin treatment	Reference values
Age (years)	27.3	27.6	
Total calcium (mmol/L)	2.2	2.3	2.1–2.5
Phosphorus (mmol/L)	1.1	1.2	0.8–1.4
Parathyroid hormone (ng/L)		53.3	15–68
Alkaline phosphatase (U/L)	454	341	35–104
25 (OH) vitamin D3 (nmol/L)	64.8	45.6	75–250
Creatinine (*µ*mol/L)	70.7	61.8	44–80
